# Prognostic Value of Systemic Inflammatory Response Markers in Patients Undergoing Neoadjuvant Chemotherapy and Gastrectomy for Advanced Gastric Cancer in the Eastern European Population

**DOI:** 10.3390/cancers14081997

**Published:** 2022-04-14

**Authors:** Agnieszka Pikuła, Magdalena Skórzewska, Zuzanna Pelc, Radosław Mlak, Katarzyna Gęca, Katarzyna Sędłak, Bogumiła Ciseł, Magdalena Kwietniewska, Karol Rawicz-Pruszyński, Wojciech P. Polkowski

**Affiliations:** 1 Department of Surgical Oncology, Medical University of Lublin, Radziwiłłowska 13 St., 20-080 Lublin, Poland; agnieszkapikula@umlub.pl (A.P.); magdalenaskorzewska@umlub.pl (M.S.); zuzannapelc@umlub.pl (Z.P.); kasiaa.geca@gmail.com (K.G.); katarzynasedlak@umlub.pl (K.S.); bogumilacisel@umlub.pl (B.C.); mag.cislo@gmail.com (M.K.); wojciechpolkowski@umlub.pl (W.P.P.); 2 Department of Human Physiology, Medical University of Lublin, Radziwiłłowska 11 St., 20-080 Lublin, Poland; radoslawmlak@umlub.pl

**Keywords:** gastric cancer, neutrophil-to-lymphocyte ratio, platelet-to-lymphocyte ratio, overall survival, neoadjuvant chemotherapy

## Abstract

**Simple Summary:**

This study aimed to verify the prognostic value of neutrophil-to-lymphocyte ratio (NLR), lymphocyte-to-monocyte ratio (LMR), and platelet-to-lymphocyte ratio (PLR) in GC patients undergoing neoadjuvant chemotherapy (NAC) and gastrectomy. Elevated NLR and PLR prior to NAC were associated with significantly higher risk of death (mOS: 36 vs. 87 months; HR = 2.21; *p* = 0.0255 and mOS: 30 vs. 87 months; HR = 2.89; *p* = 0.0034, respectively). Additionally, a significantly higher risk of death was observed in patients with elevated NLR after NAC (mOS: 35 vs. 87 months; HR = 1.94; *p* = 0.0368). Selected systemic inflammatory response markers (NLR, PLR) are significant prognostic factors in patients with advanced GC treated with NAC and gastrectomy, as shown in the Eastern European population.

**Abstract:**

The prognostic value of the systemic inflammatory response markers, namely neutrophil-to-lymphocyte ratio (NLR), lymphocyte-to-monocyte ratio (LMR), and platelet-to-lymphocyte ratio (PLR) has not yet been clarified in patients undergoing neoadjuvant chemotherapy (NAC) and gastrectomy for advanced gastric cancer (GC) in the Eastern European population. This study aimed to verify the prognostic value of NLR, PLR, and LMR in GC patients undergoing multimodal treatment. One hundred six GC patients undergoing NAC and gastrectomy between 2012 and 2020 were included. Analysed blood samples were obtained prior to NAC (pre-NAC group) and before surgical treatment (post-NAC group). To evaluate the prognostic value of the NLR, LMR, and PLR, univariable and multivariable overall survival (OS) analyses were performed. In the pre-NAC group, elevated NLR and PLR were associated with significantly higher risk of death (mOS: 36 vs. 87 months; HR = 2.21; *p* = 0.0255 and mOS: 30 vs. 87 months; HR = 2.89; *p* = 0.0034, respectively). Additionally, a significantly higher risk of death was observed in patients with elevated NLR in the post-NAC group (mOS: 35 vs. 87 months; HR = 1.94; *p* = 0.0368). Selected systemic inflammatory response markers (NLR, PLR) are significant prognostic factors in patients with advanced GC treated with NAC and gastrectomy, as shown in the Eastern European population.

## 1. Introduction

Gastric cancer (GC) remains a considerable burden of cancer-related mortality worldwide. However, the development of new biomarkers may improve its non-favourable outcome [1].

GC patients should undergo multidisciplinary treatment, including endoscopy, surgery, chemotherapy, and radiation therapy. In Europe, neoadjuvant chemotherapy (NAC) with a platinum and fluoropyrimidine combination is recommended for at least stage IB resectable GC patients [2]. The FLOT4 regimen (5-Fluorouracil, Leucovorin, Oxaliplatin, Docetaksel) is currently the preferred regimen for NAC, demonstrating improved survival in patients with resectable GC when compared to ECX/ECF (Epirubicin, Cisplatin, Capecitabine or 5-Fluorouracil) [3,4,5]. 

Personalised therapy in GC depends on the appropriate staging. Despite visible progress in computed tomography (CT) performed in specialised centres, the inadequacy of evaluating the tumour stage is a critical issue in clinical practice [6,7,8]. Staging laparoscopy enables the diagnosis of peritoneal dissemination with improved sensitivity and specificity compared to CT [7]; however, assessment of the nodal stage remains difficult [9]. There is a need for additional, cost-effective and readily available prognostic and predictive factors to select patients who may require prolonged NAC. Systemic inflammatory markers are of special interest since they are strongly correlated with the progression and response to cancer treatment [10]. These include blood count parameters, such as neutrophil, lymphocyte, monocyte and platelet number, as well as their combinations: neutrophil-to-lymphocyte ratio (NLR), lymphocyte-to-monocyte ratio (LMR), and the platelet-to-lymphocyte ratio [PLR] [11]. Among complete blood count derived markers, anaemia is recognised as an independent prognostic factor in numerous malignancies [12]. As the results of currently available studies are inconclusive, interpretation of NLR, PLR and LMR in routine practice is equivocal. Importantly, available literature provides data solely from the Asian population [13,14,15,16,17,18,19,20,21,22,23,24,25,26,27]. Exploratory analysis of the REAL-2 trial revealed that a high NLR value had a significant, independent negative prognostic effect. Although the study was conducted in the Western population, it should be noted that most patients had inoperable or metastatic oesophagogastric cancer [27].

To the best of our knowledge, this is the first study that aimed to verify the prognostic value of NLR, PLR and LMR in GC patients undergoing multimodal treatment in the Eastern European population.

## 2. Materials and Methods

**Figure 1:**
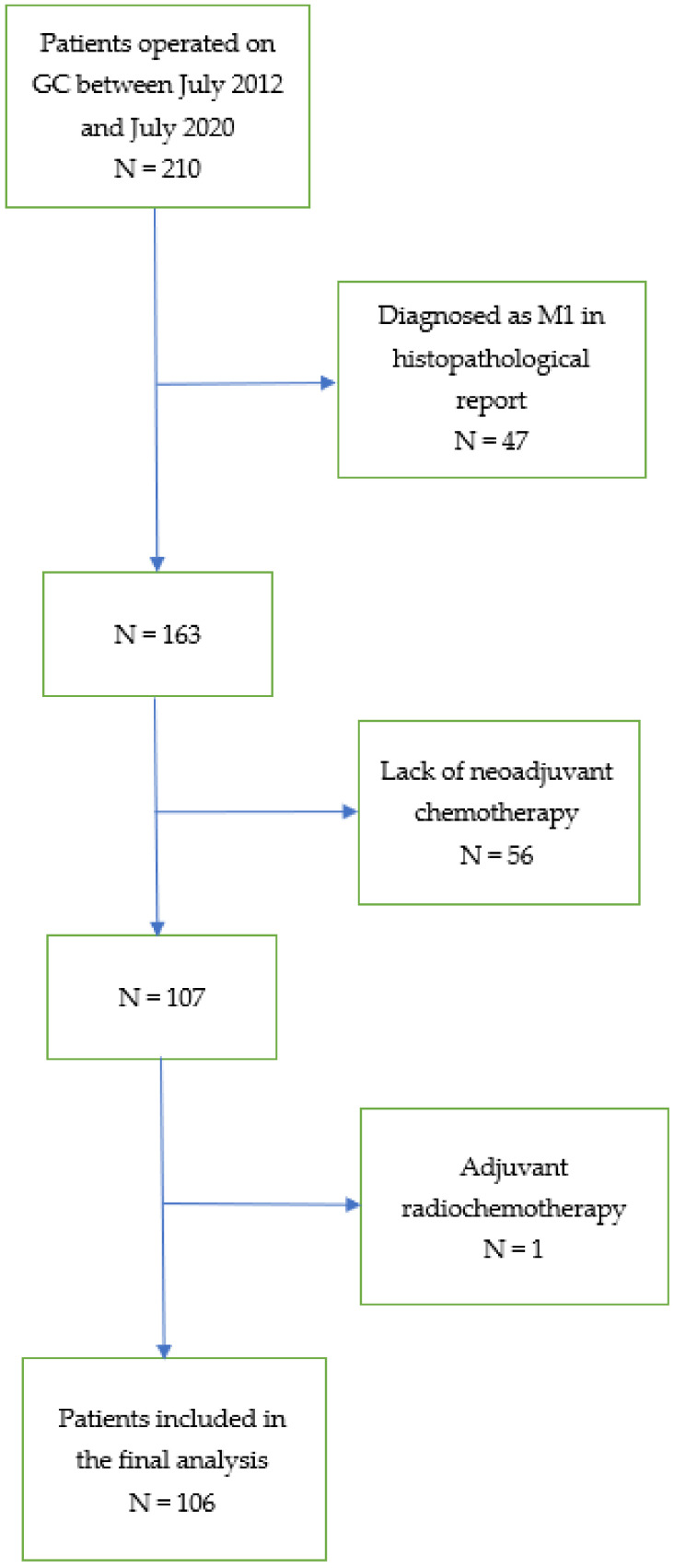
Flow chart of the study. (GC—gastric cancer; M1—distant metastases; N—number of patients).

After receiving institutional review board approval (KE—0254/331/2018), we collected data from the database of patients diagnosed with GC treated with gastrectomy from 1 July 2012 to 31 July 2020 at the Department of Oncological Surgery of the Medical University of Lublin, Poland. The initial date of patients’ recruitment was set due to the standardisation of the NAC with 5-Fluorouracil and platinum derivatives, reflecting the current evidence-based clinical guidelines for GC [2,28,29]. The preoperative staging, evaluation of the patient’s general condition and treatment plan were carried out by the multidisciplinary team. A ypTNM stage of the disease was established according to the 8th edition of the American Joint Committee on Cancer (AJCC) [30]. In the present study, we included patients with histologically confirmed GC who underwent multimodal treatment based on NAC and surgical resection. We excluded patients in clinical stages I and IV, without NAC or gastrectomy and with adjuvant radiotherapy. One hundred six patients were eligible for the final analysis. The flow chart of the study is shown in Figure 1.

### 2.1. Neoadjuvant Chemotherapy (NAC)

The majority of the study group (95%) received treatment based on a combination of platinum and fluoropyrimidine derivatives. The EOX regimen included epirubicin 50 mg/m^2^ on the 1st day of the cycle, 130 mg/m^2^ oxaliplatin on the 1st day of the cycle and 625 mg/m^2^ of capecitabine twice a day from days 1–21; cycles were repeated every 21 days (30). Three cycles EOX before surgery and three cycles after surgery. The FLOT-4 regimen consisted of docetaxel at 50 mg/m^2^ on day 1, oxaliplatin at 85 mg/m^2^ on day 1, leucovorin at 200 mg/m^2^ on day 1 and 5-fluorouracil at 2600 mg/m^2^ on day 1 of the cycle, cycles were repeated every 14 days [4]. After 4–5 week time intervals, patients were scheduled for surgical treatment.

### 2.2. Complete Blood Count Based Inflammatory Response Markers

Blood samples used for analysis were obtained one day before administration of the first NAC cycle (pre-NAC group) and one day before surgical treatment (post-NAC group). The NLR was calculated by dividing the absolute number of neutrophils by the absolute number of lymphocytes in the peripheral blood. The PLR was calculated by dividing the absolute number of neutrophils by the absolute number of lymphocytes in the peripheral blood. The LMR was calculated by dividing the absolute number of lymphocytes by the absolute number of peripheral blood monocytes.

### 2.3. Statistical Analysis

The main outcome measured was overall survival (OS). Survival data were obtained from medical records of both hospitalisation and follow-up visits. Additionally, data from the National Health Fund were analysed, as well as personal telephone information obtained from patients and/or their families during the COVID pandemic. For this research, the observation was finalised on 1 August 2021. Statistical analysis of the data was performed using MedCalc v.15.8 (MedCalc Software, Oostende, Belgium). To assess the normality of the data distribution, the D’Agostino-Pearson test was used. Since all continuous variables had non-normal data distribution, the median was used to measure data concentration and the data spread was presented by the interquartile range and minimum-maximum range. Categorised and dichotomised variables were expressed as numbers and percentages. As an objective method of cut-off establishment, the Receiver operating characteristic (ROC) analysis was used. Determined cut-offs for pre-treatment NLR, PLR and LMR were ≤1.76, ≤132.34 and >2.75, respectively, whereas these values for the preoperative phase of POC, a day before gastrectomy were ≤1.94, ≤151.06 and >2.81, respectively. OS was defined as the time from the date of surgery to the date of the patient’s death or the date of the last follow-up. The log-rank test was used to calculate the proportional hazard ratio in univariable OS analysis (the Kaplan–Meier estimation method was used for the generation of survival curves), whereas Cox logistic regression models were used in multivariable OS analysis. Comparisons of studied ratios depending on demographic and clinical variables were performed with the use of the Mann–Whitney U test (if 2 independent groups were compared) or ANOVA Kruskal–Wallis test (if more than 2 independent groups were compared). In all analyses, we used two-tailed p-tests and results with a *p*-value below 0.05 were statistically significant.

## 3. Results

### 3.1. Characteristics of the Research Group

One hundred six patients were included. The majority of the study group was male (61.3%). The median age of patients was 61 years (range 38–76 years). Patients with middle (39.6%) or upper (34%) GC were predominant. According to Lauren’s classification, the intestinal type was predominant (53.8%). Patients in the ypT4 stage accounted for 13.2% of patients. Nearly half of the patients (49.5%) were node-positive (ypN1-N3b). None of the patients had distant metastases. Fifty-five patients (52%) were treated with chemotherapy according to the EOX regimen (33), whereas 32 patients (30%) according to the FLOT-4 regimen [4]. The median number of preoperative chemotherapy cycles was 4 and the postoperative cycles were 3.5. Detailed data on the characteristics of patients in terms of demographic and clinical variables are presented in Table 1.

### 3.2. Survival Analysis

#### 3.2.1. Univariate Analysis

The relationship between clinical, demographic variables and systemic inflammatory response markers and the OS are presented in Table 2 and Table 3. 

**Figure 2:**
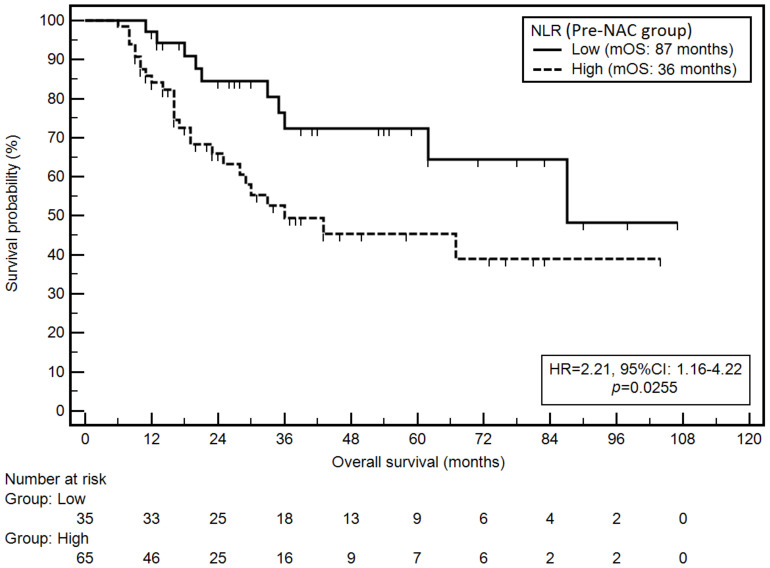
Kaplan–Meier graph representing survival probability in relation to the NLR (pre-NAC group).

Following demographic and clinical variables were associated with a significantly higher risk of death: anatomical location in esophagogastric junction (EGJ) (mOS: 11 vs. 87 months; HR = 4.70; *p* = 0.0040), lymph node metastases (N1-N3b) (mOS: 29 months vs. NR; HR = 4.43; *p* < 0.0001), grade G3 (mOS: 33 months vs. NR; HR = 2.77; *p* = 0.0038) and no pathological tumour response to NAC (TRG3, TRG4) (mOS: 35 months vs. NR; HR = 6.42; *p* = 0.0003). On the other hand, the intestinal type was associated with a significantly lower risk of death (mOS: 87 vs. 35 months; HR = 0.51; *p* = 0.0394). Elevated NLR in the pre-NAC group was associated with a significantly higher risk of death (mOS: 36 vs. 87 months; HR = 2.21; *p* = 0.0255; Figure 2). 

**Figure 3:**
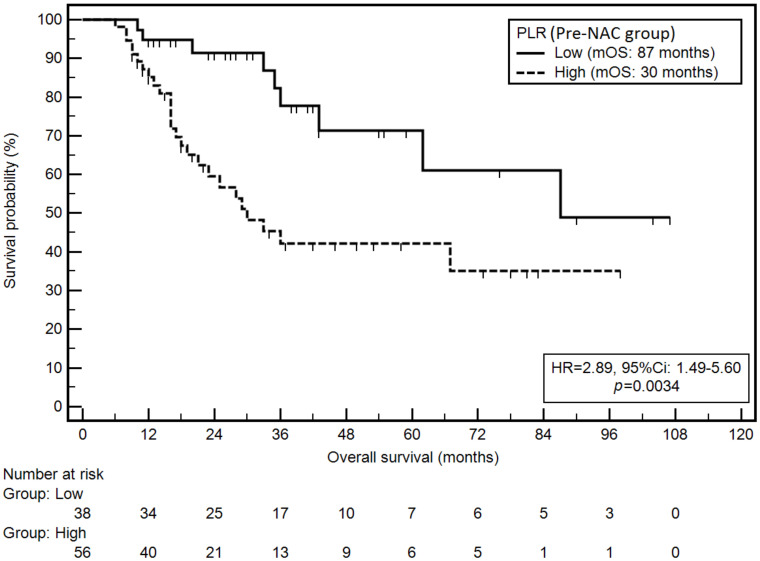
Kaplan–Meier graph representing survival probability in relation to the PLR (pre-NAC group).

Similarly, elevated PLR in the pre-NAC group was associated with a significantly higher risk of death (mOS: 30 vs. 87 months; HR = 2.89; *p* = 0.0034; Figure 3).

**Figure 4:**
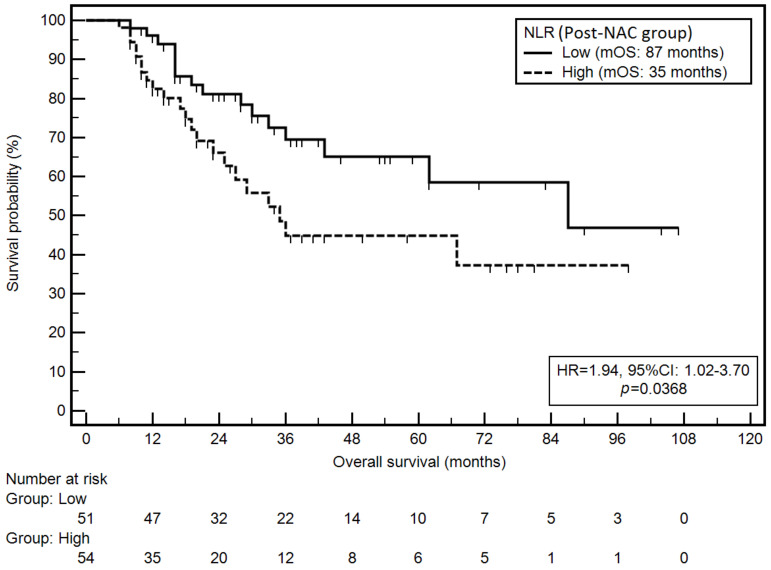
Kaplan–Meier graph representing survival probability in relation to the NLR (post-NAC group).

Moreover, high NLR in the post-NAC group was associated with a significantly higher risk of death (mOS: 35 vs. 87 months; HR = 1.94; *p* = 0.0368; Figure 4).

#### 3.2.2. Multivariate Analysis

Multivariate analysis confirmed the independent, unfavourable prognostic value of anatomical location (EGJ) (HR = 6.63; *p* = 0.0042), positive lymph nodes (HR = 3.94; *p* = 0.0009), grade G3 (HR = 2.66; *p* = 0.0304) and no pathological tumour response to NAC (TRG3, TRG4; HR = 5.92, *p* = 0.0066). 

Moreover, in multivariate analysis, elevated NLR in the pre-NAC group was associated with a non-significantly higher risk of death (HR = 1.29; *p* = 0.5263). Whereas elevated PLR in the pre-NAC group was associated with a significantly higher risk of death (HR = 2.75; *p* = 0.0143). Elevated NLR in the post-NAC group was associated with a significantly higher risk of death (HR = 1.97; *p* = 0.0414). 

### 3.3. Relationship of Demographic and Clinical Variables with the Systemic Inflammatory Response Markers 

The age of patients was the only factor related to one of the systemic inflammatory response markers. Patients under 61 years old (median, Table 1) had significantly lower LMR compared to patients over 61 years old (Me: 3.58 vs. 2.87; *p* = 0.0188). There were no statistically significant relationships between other demographic and clinical variables and the systemic inflammatory response markers.

## 4. Discussion

This study aimed to verify the prognostic value of the systemic inflammatory response markers in patients with advanced GC treated with NAC and gastrectomy. PLR in the pre-NAC group and NLR in the post-NAC group were independent prognostic factors in patients undergoing multimodal treatment. Although tumour-related neutrophils are significant factors in promoting angiogenesis, tumour growth or metastasis, the exact correlation between higher NLR values and poor prognosis remains unclear [10,31]. Recently, Asian research confirmed PLR to be an independent prognostic factor for OS also in patients undergoing adjuvant chemoradiotherapy after radical gastrectomy [32]. Moreover, the prognostic model, including systemic inflammation, was more precise than the model without systemic inflammation. 

Various original studies and meta-analyses have evaluated systemic inflammatory response markers in GC patients, as shown in Table 4.

Overviewed studies are mostly retrospective, published between 2020–2021 and conducted in the Far East (China and Japan). Moreover, all patients were treated with NAC followed by surgical resection. Notably, the rate of NAC administration in the Asian GC population is rather low when compared with the West. A meta-analysis of 28,929 patients from 51 international cohorts (10 from Europe and the USA and 41 from Asia), evaluating the role of PLR in GC patients treated with surgery and chemotherapy, concluded high PLR is associated with a shorter OS in regard to more frequent involvement of the serosa, lymph node metastases and a higher rate of stage III/IV [25]. Elevated PLR had a high prognostic impact only in the Asian population. Furthermore, considering different treatment methods, high PLR was a significant predictor of shorter OS in patients undergoing surgery alone. However, it was of no prognostic value in patients receiving chemotherapy or combined treatment [25]. Another meta-analysis of 28 studies confirmed the association between high PLR values and unfavourable OS [26]. Twenty-one studies were conducted in Asia and seven in Europe/America. Patients with elevated PLR had significantly shorter OS.

In patients undergoing gastrectomy with curative intent, increasing evidence underlines that systemic inflammatory response, triggered by higher neutrophil and platelet levels, worsens the prognosis [11]. Mohri et al. concluded that evaluation of NLR prior to the treatment remains the crucial host-related parameter affecting prognosis in patients with GC [33], possibly due to intracellular instability through atypical cell proliferation leading to further deterioration of the cell’s environment [34]. Our study confirms the prognostic value of these markers in the Caucasian population. Elevated NLR and PLR values were associated with a higher risk of death.

A Chinese study aimed to verify the prognostic relationship between NAC and conversion rates of NLR and PLR in patients with unresectable and metastatic GC. Patients with low inflammatory response markers rates after NAC had significantly longer progression-free survival when compared to patients with sustained high-NLR/PLR [35]. In our study, patients with poor or no response to NAC (Tumour Regression Grade 3 and 4) had a significantly higher risk of death in both univariable and multivariable analyses.

Limitations of our study include its retrospective and single-centre nature and relatively small sample size. A prospective multi-centre study with a large sample size would overcome these shortcomings and possibly validate our current findings.

## 5. Conclusions

Selected systemic inflammatory response markers (NLR, PLR) are significant prognostic factors in patients with advanced GC treated with NAC and gastrectomy, as shown in the Eastern European population.

## Figures and Tables

**Table 1:** Demographic and clinical characteristics of the study group.

Variable	No. of Patients n = 106 (%)
**Sex**	
Female	41 (38.7%)
Male	65 (61.3%)
**Age (years)**	
Median	61
Min–Max	38–76
**Anatomical localisation of gastric cancer**	
Esophagogastric junction	4 (3.8%)
Upper	36 (34.0%)
Middle	42 (39.6%)
Lower	24 (22.6%)
**Lauren’s histological type**	
Intestinal	56 (53.8%)
Diffuse	14 (13.5%)
Mixed	34 (32.7%)
Missing data (n = 2)	
**ypT**	
T0	6 (5.7%)
T1a	5 (4.7%)
T1b	9 (8.5%)
T2	29 (27.4%)
T3	43 (40.6%)
T4a	12 (11.3%)
T4b	2 (1.9%)
**ypN**	
N0	53 (50.5%)
N1	14 (13.3%)
N2	17 (16.2%)
N3a	10 (9.5%)
N3b	11 (10.5%)
Missing data (n = 1)	
**ypM**	
M0	106 (100%)
**Grading**	
G1	6 (6.1%)
G2	43 (43.9%)
G3	49 (50.5%)
Missing data (n = 8)	
**No. of neoadjuvant chemotherapy cycles**	
Median	4
Min–Max	1–6
**Adjuvant chemotherapy compliance**	
Yes	101 (95.3%)
No	5 (4.7%)
**Tumour regression grade**	
1	7 (6.7%)
2	28 (26.7%)
3	48 (45.7%)
4	22 (21.0%)
Missing data (n = 1)	

**Table 2:** Relationship between the systemic inflammatory response markers on overall survival.

Variable	Pre-NAC Group			Post-NAC Group		
	Univariate		Multivariate ^#^	Univariate		Multivariate ^#^
	mOS	*p* HR [95%CI]	*p* HR [95%CI]	mOS	*p* HR [95%CI]	*p* HR [95%CI]
**NLR**						
Low	87.00	0.0255 *	0.5263	87.00	0.0368 *	0.0414 *
High	36.00	2.21 [1.16–4.22]	1.29 [0.59–2.80]	35.00	1.94 [1.02–3.70]	1.97 [1.03–3.76]
**PLR**						
Low	87.00	0.0034 *	0.0143 *	87.00	0.5872	0.8887
High	30.00	2.89 [1.49–5.60]	2.75 [1.23–6.13]	67.00	1.21 [0.59–2.49]	0.95 [0.44–2.03]
**LMR**						
Low	-	0.7495	0.5312	87.00	0.8035	0.6845
High	67.00	0.89 [0.43–1.86]	1.28 [0.59–2.76]	62.00	0.92 [0.47–1.79]	1.16 [0.57–2.37]

CI—confidence interval, HR—hazard ratio, LMR—lymphocyte to monocyte ratio, mOS—median overall survival, POC- perioperative chemotherapy, NLR—neutrophil to lymphocyte ratio, PLR—platelet to lymphocyte ratio. *—statistically significant results. #—in multivariate analysis, the results were adjusted for all statistically significant results of univariate analysis (including both Table 2 and Table 3).

**Table 3:** Influence of selected demographic and clinical variables on overall survival.

Variable	Univariable		Multivariable ^#^
	mOS	*p* HR [95%CI]	*p* HR [95%CI]
**Sex**			
Female	62	0.4674	0.5751
Male	87	0.78 [0.40–1.54]	0.81 [0.39–1.67]
**Age (years)**			
< Median	87	0.9684	0.4542
>=Median	62	1.01 [0.533–1.91]	1.31 [0.5–2.63]
**Anatomical localisation**			
Oesophagogastric junction	11	0.0040 *	0.0042 *
Non–junctional	87	4.70 [0.43–51.84]	6.43 [1.81–22.80]
**Lauren’s histological type**			
Intestinal	87	0.0394 *	0.756
Diffuse, Mixed	35	0.51 [0.27–0.98]	0.88 [0.39–1.97]
**ypT**			
T0–T3	87	0.4898	0.5101
T4a–T4b	62	1.35 [0.51–3.58]	1.38 [0.53–3.60]
**ypN**			
N0	NR	<0.0001 *	0.0009 *
N1–N3b	29	4.43 [2.33–8.44]	3.94 [1.76–8.84]
**Grade**			
G1–G2	NR	0.0038 *	0.0304 *
G3	33	2.77 [1.45–5.27]	2.66 [1.10–6.44]
**Tumour regression grade**			
1–2	NR	0.0003 *	0.0066 *
3–4	35	6.42 [3.30–12.48]	5.92 [1.65–21.25]

CI—confidence interval, HR—hazard ratio, NR—not reached, mOS—median overall survival, *—statistically significant results. **#**—in multivariable analysis, the results were adjusted for all statistically significant results of univariable analysis (including Table 2).

**Table 4:** Baseline characteristics of the selected studies in comparison to the results of the present study.

Study	Publication Year	Country	Study Design	Treatment Strategy	Stage	Sample Size	Significant Prognostic Blood Markers
Yin [13]	2021	China	retrospective	Surgery AC when applicable	I, II, III	852	SII, PLR
Sun [14]	2020	China	retrospective	-	-	488	NLR, PLT, PDW, ABO blood group
Lin [15]	2020	China	retrospective	Surgery AC when applicable	I, II, III	1810	LMR, Hg,
Lin [16]	2021	China	retrospective	Surgery AC when applicable	I, II, III	2257	LMR
Xu [17]	2020	China	prospective	Surgery AC when applicable	I, II, III	438	LMR
Nakamura [18]	2020	Japan	retrospective	NAC SurgeryAC	IV	50	NLR, albumin levels
Gu [19]	2020	China	prospective	Surgery AC	II, III	598	PLR
Kudou [20]	2020	Japan	retrospective	Surgery AC when applicable	I, II, III	206	GPS, PI and PNI
Hirahara [21]	2020	Japan	retrospective	Surgery AC when applicable	I, II, III	412	SII
Toyokawa [22]	2020	Japan	retrospective	Surgery AC	III	225	CAR, PLR
Liu [23]	2021	China	retrospective	Surgery AC when applicable	I, II, III	442	SIRI
Ohe [24]	2020	Japan	retrospective	NAC Surgery AC	III, IV	41	PLR
present study	-	Poland	retrospective	NAC Surgery	II, III	106	NLR, PLR

AC—adjuvant chemotherapy, NAC—neoadjuvant chemotherapy (NAC + AC = POC), SII—systemic immune-inflammation index, SII = N × P/L (N = Neutrophil count, L = Lymphocyte count and P = Platelet count), PDW—platelet distribution width, PI—prognostic index, PI ranges based on the CRP level and the white blood cell count, PNI—prognostic nutritional index, PNI was calculated using the following formula: 10 × serum albumin (g/dL) + 0.005 × total lymphocyte count (per mm^3^), GPS—Glasgow prognostic score, GPS ranges based on CRP and albumin level, SIRI—systemic inflammation response index, SIRI = neutrophil count × monocyte count/lymphocyte count, CAR—C-reactive protein/ albumin ratio.

## Data Availability

Data available on special request.
